# Supplementary material for: Multiple Criteria Optimization (MCO): A gene selection deterministic tool in RStudio
Source: PLoS One. 2022 Jan 27;17(1):e0262890. doi: 10.1371/journal.pone.0262890 (PMC8794188; doi:10.1371/journal.pone.0262890)
Supplement: S1 File — (ZIP) [file pone.0262890.s001.zip › Supporting Information.pdf]

Supporting Information- Multiple Criteria Optimization (MCO): a gene selection deterministic tool in RStudio

Isis Narváez-Bandera[1], Deiver Suárez-Gómez[1], Clara E. Isaza[1,2,3,4], Mauricio Cabrera-Ríos[1,5*]

1 Bioengineering Graduate Program, The Applied Optimization Group, University of Puerto Rico-Mayagüez, Mayagüez 00681, Puerto Rico
2 Public Health Program, Ponce Health Sciences University, Ponce 00732, Puerto Rico
3 Basic Sciences Department, Ponce Health Sciences University, Ponce 00732, Puerto Rico
4 Biology Department, University of Puerto Rico-Mayagüez, Mayagüez 00681, Puerto Rico
5 Industrial Engineering Department, The Applied Optimization Group, University of Puerto Rico-Mayagüez, Mayagüez 00681, Puerto Rico

* mauricio.cabrera1@upr.edu

Comparative Study: MCO Vs CFS, IG and eBayes gene selection methods

In this section, a comparative study on classification accuracy performance, dispersion, and parsimony was performed, including MCO and three well-established gene selection methods: Correlation-based Feature Selection (CFS) [1], Information Gain (IG) [2], and eBayes [3]. These methods were selected based on their ability to work with large data dimensionality:

- Correlation-based Feature Selection (CFS) Assesses a subset of genes depending on a correlation measure with the class according to a heuristic search strategy such as greedy hill climbing and best first [1]. It is expected that genes within the selected subsets meet the following two characteristics: first, to be highly predictive for the class, and second to have no correlation between them. This study selected every subset with a complete set and with 10-fold cross-validation (CV) form using WEKA software [4].
- Information Gain (IG) IG is the expected reduction in entropy on the groups formed by the genes. It gives an ordered ranking for all the genes comparing the entropy before and after one split, and a threshold -selected by the analyst- is needed for choosing those genes with the highest information gain [2]. In this study, IG was implemented using the information.gain function from FSelector R package [5]. For each dataset, whenever possible, four cutoff values were evaluated according to the weights obtained.
- eBayes: Given a linear model, compute t-statistic, F-statistic, and log-odds of differential expression by empirical Bayes moderation of the standard errors towards a common value [3]. For the implementation, the eBayes function from limma R package [6] was used.

Classification accuracy was measured through five classification methods, which commonly differ in results: Support Vector Machines (SVM) [7], K- Nearest Neighbors (KNN) [8], treebag [9], Linear Discriminant Analysis (LDA) [10] and Random Forest (RFs) [11]. To improve the model error estimate, a 10-fold CV was performed in all classifiers. The trainControl function from caret R package [12] was used for CV purposes. We used the method "repeatedcv" as well as 10 for the number of folds or number of resampling iterations and 3 for the repeats parameter. Finally, fitting predictive models was performed with the train function from the caret R package with different tuning parameters. Specifically, for the SVM implementation, we used the tuneGrid parameter for variation of the sigma value (from 0 to 0.9) and the C regularization constant (from 0 to 2.5). Similarly, for the RF implementation, we used the tuneGrid parameter for variation of the mtry value. For KNN, LDA, and treebag, the train function was used with default parameters. Finally, MCO was carried out using ten frontiers, as advocated by our research group. The four gene selection methods were then assessed in three responses: classification accuracy, classification dispersion, and parsimony of the final selection (number of genes selected by the method). These three responses were assessed across the five classification methods, and parameter variation for each method, using the four databases for Parkinson´s Disease presented in the main body of the manuscript (GSE99039, GSE18838, GSE19587, and GSE57475) (Supplementary Table 1).

In terms of classification accuracy (Supplementary Fig 1), an ANOVA [13] deemed that at least one of the gene selection methods had a different classification accuracy mean (p-value = 0.003). Furthermore, using Tukey´s multiple comparison scheme [14], it was determined that the CFS method provided the largest classification accuracy. At the same time, the other three (MCO, IG, and eBayes) showed no significant difference between them at an alpha value of 0.05. This provides evidence of the competitiveness of MCO with the distinctive characteristic that no parameters need to be adjusted by the user.

Regarding classification accuracy variance (dispersion) (Supplementary Fig 2), there was evidence that at least one of the methods had a different variance than the rest (p-values of 0.012 and 0.090 for Multiple Comparison´s and Levene´s methods [15] respectively). Furthermore, an inspection of the confidence intervals determined that MCO´s interval did not overlap with the other three method's intervals and that MCO´s interval contained lower values than the rest of the methods. This evidences MCO´s lower results´ dispersion and, therefore, its robust classification performance across different classifiers and datasets.

Finally, regarding the number of selected genes (parsimony) (Supplementary Fig 3), an ANOVA [13] showed that at least one of the methods had a different mean number of selected genes (p-value = 0.011). Furthermore, using Tukey´s multiple comparison scheme [14], two methods were deemed statistically equivalent in their number of selected genes: MCO and CFS, both of which showed a significantly lower mean number of selected genes than eBayes. The IG method did not show a significant difference with CFS and eBayes due to its large dispersion. This result evidences the parsimonious gene selection qualities of MCO.

In conclusion, this comparison shows the robustness of MCO solutions, the results of which tend to be parsimonious, providing a competitive classification accuracy performance without requiring the adjustment of statistical or computational parameters and with the unique capability to support simultaneous meta-analysis of multiple datasets.

Table 1. Gene selection techniques through classification methods

Classifiers	FS	57475: 101 Samples Training	# predictors	Accuracy:	18838: 20 Samples Training	# predictors	Accuracy:	19587: 16 Samples Training	# predictors	Accuracy:	99039: 308 Samples Training	# predictors	Accuracy:
SVM	MCO	10 Frontier	42	0.731707317	10 Frontier	68	0.75	10 Frontier	47	0.6666667	10 Frontier	24	0.5846154
	CFS	Full	24	0.707317073	Full	62	0.875	Full	69	0.83333	Full	98	0.7076923
		CV_10fold	123	0.80487	CV_10fold	311	0.75	CV_10fold	278	0.8333	CV_10fold	417	0.6384
	IG	Corr: 0.05	27	0.707317073	Corr:0.4	36	0.5	Corr:0.6	4	0.66666	Corr:0.035	22	0.6230769
			NA	NA	Corr:0.3	142	0.75	Corr:0.5	14	0.83333	Corr:0.03	65	0.6076923
			NA	NA	Corr:0.2	1444	0.625	Corr:0.4	31	1	Corr:0.02	477	0.6307692
			NA	NA	Corr:0.1	1573	0.625	Corr:0.3	336	0.666666	Corr:0.01	523	0.6230769
	ebayes	PV:0.99998	25143	stack overflow	PV:0.09	127	0.75	PV:0.23	267	0.83333	PV:0.01	350	0.6153846
			NA	NA	PV:0.1	276	0.75	PV:0.24	309	0.66666	PV:0.05	954	0.5307692
			NA	NA	PV:0.15	1439	0.625	PV:0.25	346	0.666666	PV:0.09	1425	0.5307692
			NA	NA	PV:0.2	2494	0.625	PV:0.26	1317	0.5	PV:0.1	1497	0.5307692
KNN	MCO		42	0.609756098	10 Frontier	68	0.5	10 Frontier	47	0.5	10 Frontier	24	0.5692308
	CFS	Full	24	0.756097561	Full	62	0.625	Full	69	0.8333333	Full	98	0.6615385
		CV_10fold	123	0.658536585	CV_10fold	311	0.5	CV_10fold	278	0.8333333	CV_10fold	417	0.6846154
	IG	Corr: 0.05	27	0.658536585	Corr:0.4	36	0.5	Corr:0.6	4	0.5	Corr:0.035	22	0.6230769
			NA	NA	Corr:0.3	142	0.5	Corr:0.5	14	0.5	Corr:0.03	65	0.5769231
			NA	NA	Corr:0.2	1444	0.5	Corr:0.4	31	0.833333	Corr:0.02	477	0.5923077
			NA	NA	Corr:0.1	1573	0.5	Corr:0.3	336	0.5	Corr:0.01	523	0.6
	ebayes	0.99998	25143	stack overflow	PV:0.09	127	0.5	PV:0.23	267	0.6666667	PV:0.01	350	0.5538462
			NA	NA	PV:0.1	276	0.5	PV:0.24	309	0.6666667	PV:0.05	954	0.6538462
			NA	NA	PV:0.15	1439	0.5	PV:0.25	346	0.6666667	PV:0.09	1425	0.6
			NA	NA	PV:0.2	2494	0.5	PV:0.26	1317	0.6666667	PV:0.1	1497	0.6461538
treebag	MCO		42	0.56097561	10 Frontier	68	0.625	10 Frontier	47	0.5	10 Frontier	24	0.6384615
	CFS	Full	24	0.682926829	Full	62	0.5	Full	69	0.6666667	Full	98	0.6923077
		CV_10fold	123	0.780487805	CV_10fold	311	0.375	CV_10fold	278	0.6666666	CV_10fold	417	0.7
	IG	Corr: 0.05	27	0.731707317	Corr:0.4	36	0.75	Corr:0.6	4	1	Corr:0.035	22	0.6230769
			NA	NA	Corr:0.3	142	0.375	Corr:0.5	14	0.6666667	Corr:0.03	65	0.6307692
			NA	NA	Corr:0.2	1444	0.5	Corr:0.4	31	0.8333333	Corr:0.02	477	0.6538462
			NA	NA	Corr:0.1	1573	0.5	Corr:0.3	336	0.6666667	Corr:0.01	523	0.6153846
	ebayes	PV:0.99998	25143	stack overflow	PV:0.09	127	0.375	PV:0.23	267	0.8333333	PV:0.01	350	0.6461538
			NA	NA	PV:0.1	276	0.375	PV:0.24	309	0.8333333	PV:0.05	954	0.6153846
			NA	NA	PV:0.15	1439	0.625	PV:0.25	346	0.8333333	PV:0.09	1425	0.6153846
			NA	NA	PV:0.2	2494	0.5	PV:0.26	1317	0.8333333	PV:0.1	1497	0.6153846
LDA	MCO		42	0.536585366	10 Frontier	68	0.625	10 Frontier	47	0.6666667	10 Frontier	24	0.661538
	CFS	Full	24	0.682926829	Full	62	0.875	Full	69	0.8333333	Full	98	0.61538
		CV_10fold	123	0.536585366	CV_10fold	311	0.875	CV_10fold	278	1	CV_10fold	417	0.6
	IG	Corr: 0.05	27	0.6585366	Corr:0.4	36	0.625	Corr:0.6	4	1	Corr:0.035	22	0.615384
			NA	NA	Corr:0.3	142	0.5	Corr:0.5	14	0.1666667	Corr:0.03	65	0.653846
			NA	NA	Corr:0.2	1444	0.5	Corr:0.4	31	0.5	Corr:0.02	477	0.6615384
			NA	NA	Corr:0.1	1573	0.5	Corr:0.3	336	0.8333333	Corr:0.01	523	0.638461
	ebayes	PV:0.99998	25143	stack overflow	PV:0.09	127	0.75	PV:0.23	267	0.8333333	PV:0.01	350	0.5153846
			NA	NA	PV:0.1	276	0.75	PV:0.24	309	0.8333333	PV:0.05	954	0.6846154
			NA	NA	PV:0.15	1439	0.5	PV:0.25	346	0.8333333	PV:0.09	1425	0.5538462
			NA	NA	PV:0.2	2494	0.5	PV:0.26	1317	0.8333333	PV:0.1	1497	0.6923077
RF	MCO		42	0.658536585	10 Frontier	68	0.625	10 Frontier	47	0.6666667	10 Frontier	24	0.59231
	CFS	Full	24	0.780487805	Full	62	1	Full	69	1	Full	98	0.69231
		CV_10fold	123	0.731707317	CV_10fold	311	1	CV_10fold	278	1	CV_10fold	417	0.7461538
	IG	Corr: 0.05	27	0.78049	Corr:0.4	36	0.625	Corr:0.6	4	1	Corr:0.035	22	0.6230769
			NA	NA	Corr:0.3	142	1	Corr:0.5	14	1	Corr:0.03	65	0.6461538
			NA	NA	Corr:0.2	1444	0.7222222	Corr:0.4	31	0.8333333	Corr:0.02	477	0.6923077
			NA	NA	Corr:0.1	1573	0.7111111	Corr:0.3	336	0.8333333	Corr:0.01	523	0.6923077
	ebayes	PV:0.99998	25143	stack overflow	PV:0.09	127	1	PV:0.23	267	0.8333333	PV:0.01	350	0.6769231
			NA	NA	PV:0.1	276	0.875	PV:0.24	309	0.8333333	PV:0.05	954	0.7
			NA	NA	PV:0.15	1439	0.625	PV:0.25	346	0.8333333	PV:0.09	1425	0.6769231
			NA	NA	PV:0.2	2494	0.5	PV:0.26	1317	0.8333333	PV:0.1	1497	0.6846154

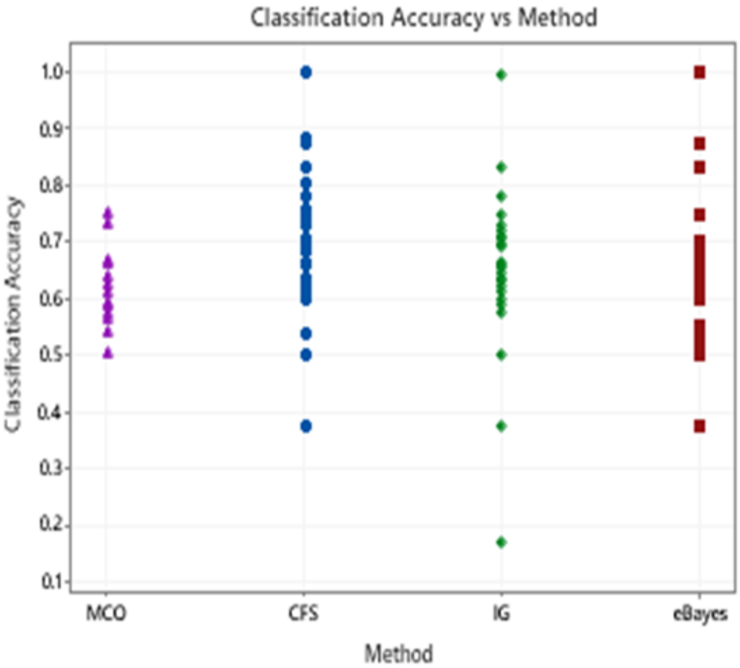

Analysis of Variance

Source	DF	Adj SS	Adj MS	F-Value	P-Value
Method	3	0.2968	0.09894	4.92	0.003
Error	181	3.6430	0.02013		
Total	184	3.9399			

Grouping Information Using the Tukey Method and 95% Confidence

Method	N	Mean	Grouping	
CFS	40	0.7446	A	
eBayes	60	0.6605		B
IG	65	0.6599		B
MCO	20	0.6134		B

Means that do not share a letter are significantly different.

Fig 1. ANOVA of Classification accuracy vs. gene selection methods showed that at least one of them had a different classification accuracy mean. Tukey´s method determined that the CFS provided the largest classification accuracy, while MCO, IG, and eBayes no had significant difference between them at an alpha value of 0.05.

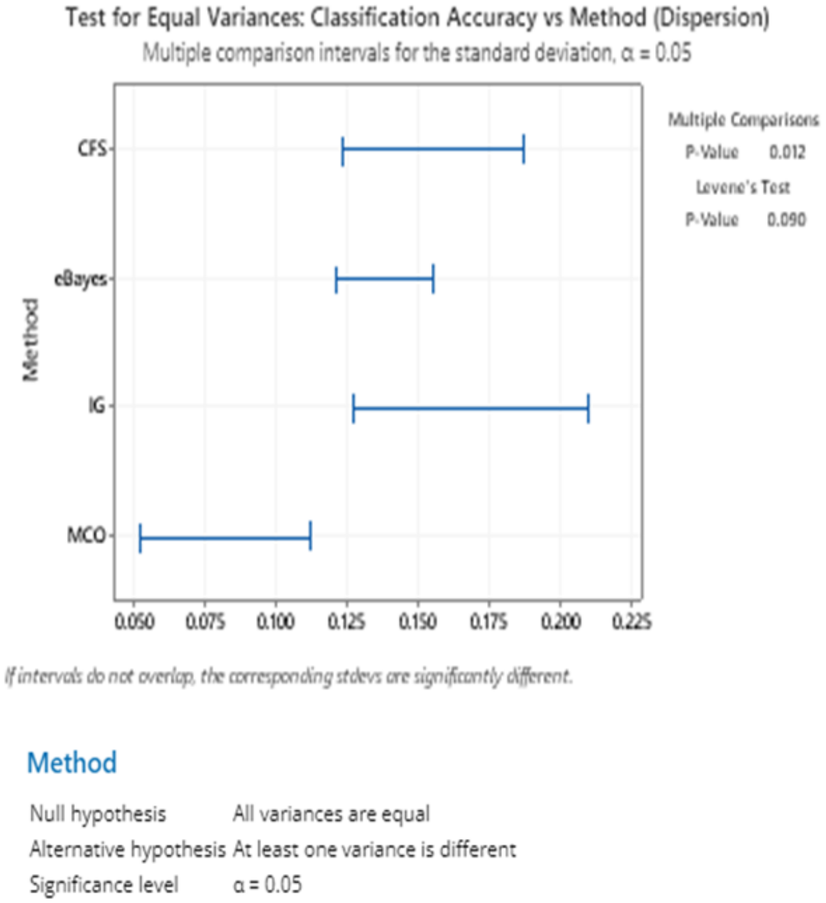

Fig 2. Test for Equal Variances of Classification accuracy vs. gene selection methods showed that at least one of them had a different variance. Multiple Comparisons and Levene´s test determined that MCO´s interval contained lower values than the other methods.

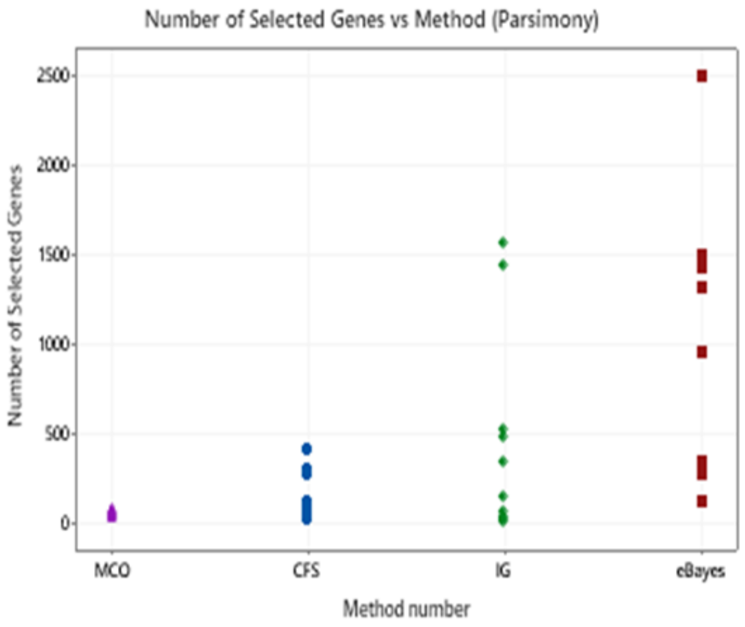

Analysis of Variance

Source	DF	Adj SS	Adj MS	F-Value	P-Value
Method	3	3799427	1266476	4.34	0.011
Error	33	9637132	292034		
Total	36	13436559			

Tukey Pairwise Comparisons

Grouping Information Using the Tukey Method and 95% Confidence

Method	N	Mean	Grouping	
eBayes	12	900	A	
IG	13	361	A	B
CFS	8	172.8		B
MCO	4	45.25		B

Means that do not share a letter are significantly different.

Fig 3. ANOVA of the number of selected genes vs. gene selection methods showed that at least one of them had a different selected genes number mean. Tukey´s method determined that MCO and CFS showed a significantly lower mean number of selected genes.

Supplementary Table 2. Results from 10 frontiers for MCO 1 thru MCO 4 pertaining to analysis instance 1-2D-9039

	Frontier	# of Genes	Gene Name					
MCO 1	1	1	TUBB2A					
	2	4	APOBEC3B	HLA	DRB4	MMP9		
	3	2	FAM46C	FECH				
	4	3	ANXA3	GPR97	KRT1			
	5	4	BPGM	HBD	HBG1	SELENBP1		
	6	5	CFD	EPB42	HBZ	SLC4A1	TRIM58	
	7	6	FFAR2	LOC731424	LY96	PGLYRP1	RPL36A	TSPAN5
	8	10	CSF3R	DDX11L2	FLOT2	GLRX5	HSPA6	PRKCD
			PTGDS	SNCA	TYMP	ZYX		
	9	9	CDA	LOC100134822	NUDT4	PADI4	PRR24	RNF24
			SLC14A1	TBC1D4	TNS1			
	10	7	AP5B1	CA4	CST7	GPR146	LRRN3	SPI1
			XK					
MCO 2	1	1	TUBB2A					
	2	1	RPL36A					
	3	4	BCL2A1	GPX3	HLA.DQB1	RP3.507I15.1		
	4	10	A2M.AS1	COX16	FOSL2	RP11.28F1.2	PLGLB1	HAT1
			PAM	IL18R1	HLA.DQA1	CSTA		
	5	9	CFD	NUDT21	SPON2	LOC100509457	RNF19B	FOS
			KIR2DS2	MYOM2	PTGDS			
	6	11	CPA3	EMC2	FKBP1A	HSPE1.MOB4	LRRN3	MKLN1
			PRF1	RP13.258O15.1	RPS11	SIPA1L2	VMP1	
	7	12	ANP32E	KLF10	KIR2DL2	CTC.510F12.4	NDUFS5	TCEAL8
			HNRNPH2	KIR2DL3	MAD1L1	NSUN6	TMCC3	ANXA3
	8	12	IFIT1	SMIM15	GPR97	SARNP	CLIC3	PRKCD
			AF001548.5	MMP9	TMEM170B	KLRF1	TATDN1	UQCRB
	9	14	ANKRD36B	APOBEC3B	ARPP19	BMX	BOLA2	C5AR1
			GSKIP	MAP2K2	KIR3DS1	PGLYRP1	NFAT5	GZMK
			SPI1	EPB41L3				
	10	15	AMFR	APOBR	C5orf28	LOC100129550	FKBP8	H1FX
			PPP1R15A	RP11.158G18.1	PPIG	LOC100127972	PSMA4	EIF4A1
			SUCLA2	KRT23	RPL9			
MCO 3	1	1	RPS4Y1					
	2	1	XIST					
	3	1	KDM5D					
	4	1	EIF1AY					
	5	1	TXLNGY					
	6	1	PRKY					
	7	2	DDX3Y	HLA.DQA1				
	8	2	HLA.DRB4	USP9Y				
	9	2	RPL22L1	UTY				
	10	3	BCL2A1	EIF1AX	PSMC6			
MCO 4	1	1	RPS4Y1					
	2	1	XIST					
	3	1	KDM5D					
	4	1	EIF1AY					
	5	2	TXLNGY	RPL36A				
	6	1	PRKY					
	7	2	DDX3Y	RP3.507I15.1				
	8	3	COX16	RPL22L1	USP9Y			
	9	4	APOBEC3B	HAT1	PRKX	TRAT1		
	10	4	COX7C	EIF1AX	SUB1	TSIX		

Supplementary Table 3. Results from 10 frontiers for MCO 1 thru MCO 4 pertaining to analysis instance 1-2D-8838

	Frontier	# of Genes	Gene Name					
MCO 1	1	1	RPS24					
	2	1	DYNLL1					
	3	4	GAFA3	HSPA1A	RAP1B	TUBA1B		
	4	3	CALM2	HIST1H4C	ND6			
	5	2	DDX3Y	S100A8				
	6	2	GTF2B	HSP90AA1				
	7	3	RPL18	TAGLN2	THYN1			
	8	3	C14orf166	TMEM14B	TMEM14E			
	9	5	GABARAP	HNRNPA1	LIMS1	PIGY	RPS4Y1	
	10	3	ANP32B	HIST1H1E	KIAA0776			
MCO 2	1	3	DND1	RPL18	TMEM14B			
	2	3	GABARAP	PI3	RPS24			
	3	4	GTF2B	NPRL3	RPSA	UBC		
	4	7	ALAS2	AQP9	EIF3L	NSUN5P2	SEPN1	SH3BGRL3
			THYN1					
	5	9	ARHGDIB	HIST1H3I	MAP2K3	MPP1	ND6	PDZK1IP1
			TAGLN2	UQCRFS1	RPL35A			
	6	4	BCL2L1	GRN	OCM2	SLC4A1		
	7	8	DAZAP2	DYNLL1	HIST1H1E	PHOSPHO1	PGK1	NADK
			TCEAL3	SELPLG				
	8	11	CEACAM4	CORO1C	FCER1G	HSPA8	LSM3	MARCH2
			OR10G9	PPT1	SELENBP1	SIRPA	NAPA	
	9	7	BSG	CD3E	GAFA3	PSMF1	SESN3	STK17B
			XPO6					
	10	11	ATP5H	CTSD	EIF4B	FERMT3	FKBP8	GPR141
			KRTAP5.3	MGAM	RAB5B	STX11	IL32	
MCO 3	1	3	HLA.DRB1	HLA.DRB5	TMEM176B			
	2	3	NPRL3	LOC100510059	TMEM176A			
	3	4	CHI3L1	HIST3H2BB	MBNL3	XAF1		
	4	3	CYBRD1	LCE1D	PRY			
	5	5	CX3CR1	CYP4F3	EMR2	PI3	TUBA3E	
	6	8	AOAH	APOB48R	AQP9	CEACAM4	FAM46C	FBXO7
			RPL35A	FCGR3A				
	7	5	CPN1	KIAA1324	PGRMC1	RIOK3	TMBIM1	
	8	8	ADIPOR1	CSDA	GRAP	KRTAP19.5	MME	OR2T12
			SIRPA	PELI1				
	9	7	GNS	GPR20	ISCA1	NADK	PROK2	RALB
			SLC25A37					
	10	12	C3orf19	CCR1	FECH	HIST1H1D	MICAL2	PTPRJ
			RNASE2	RPL35	SEC14L1	SRSF9	TCL1A	RBX1
MCO 4	1	4	DYNLL1	HIST1H4C	RAP1B	RPS24		
	2	4	HSPA1A	RPL11	S100A8	TUBA1B		
	3	4	CALM2	HSP90AA1	MYL6	S100A9		
	4	4	ANP32B	B2M	DND1	NSUN5P2		
	5	4	HLA.DRB1	LYPLA2	RPSA	THYN1		
	6	6	C11orf48	GAFA3	HNRNPA1	MARCH2	NDUFA1	NONO
	7	7	C14orf166	CD3G	EI24	GIMAP7	OAZ1	PIGY
			RPS29					
	8	8	HLA.DRB5	LOC100131541	FAM58A	HIST1H2AC	CISD1	CD14
			UFD1L	RPS23				
	9	8	DDX3Y	EIF4A1	LIMS1	HIST2H2AB	NPC2	SELL
			TMEM14E	TAOK3				
	10	7	CLEC18C	DDT	DNAJC3	UQCRFS1	RPS4Y1	UBC
			IGJ					

Supplementary Table 4. Results from 10 frontiers for MCO 1 thru MCO 4 pertaining to analysis instance 1-2D-9587

	Frontier	# of Genes	Gene Name					
MCO 1	1	1	TUBA1A					
	2	5	HBA1	HBB	PAQR6	PTPRO	RPL37A	
	3	7	GFAP	MT2A	PLEKHB1	RPL34	RPS15	RPS27
			RPS27A					
	4	4	CFL1	RPL27A	SEPP1	ZNF160		
	5	4	ATP5E	FTL	RNU86	RPS11		
	6	3	FTH1P5	RPL37	RPL9			
	7	3	OPHN1	RP5.882O7.1	TMSB10			
	8	7	DPYSL2	MT1HL1	RPL13A	RPS12	RPS18	TUBA1B
			TUBB2A					
	9	5	GAPDH	RPL41	RPS10	RPS3A	RPSA	
	10	8	EEF1G	GPM6B	HAUS2	NDRG1	PPIA	RPL23A
			TUBA1C	RPL39				
MCO 2	1	1	TUBA1A					
	2	4	MBP	TUBA1B	ZNF160	ZNF721		
	3	4	HAUS2	PAQR6	PTPRO	RP11.403P17.4		
	4	4	OPHN1	PTGDS	RPL41	RPS15		
	5	2	CFL1	PRR11				
	6	1	TUBA1C					
	7	6	APOD	FTH1	GAPDH	RP5.882O7.1	RNU86	HBA
	8	4	CNP	CRYAB	EEF1A1	FBXW12		
	9	4	APLP1	ATP5E	FKSG49	NDRG1		
	10	4	ACTB	DBNDD2	FTH1P5	PGF		
MCO 3	1	3	MBP	PAQR6	RPL23A			
	2	4	CNP	GFAP	HBA1	HBB		
	3	7	ACTB	GPM6B	MT2A	PTGDS	PTPRO	RPL34
			TMSB4X					
	4	4	CFL1	RPS27A	TUBA1B	XIST		
	5	5	APLP1	FTH1P5	RPL23A	RPS27	TF	
	6	6	ATP1B1	DPYSL2	RPS13	PLEKHB1	RPL27A	SERPINA3
	7	9	DBNDD2	FTH1	GAPDH	MT1HL1	PLP1	RPL37
			TUBA1C	RPS29	RPS15			
	8	4	FTL	NDRG1	RPL27	TMSB10		
	9	8	ACTB	ATP5E	CD74	RP5.882O7.1	NPIPA1	CLU
			RPS24	RPL39				
	10	7	EFHD1	ITM2C	MORF4L2	NGFRAP1	RPL7	RPS11
			RPS17					
MCO 4	1	4	CFL1	PTPRO	RPL37A	TUBA1A		
	2	4	ATP5E	GFAP	RPL37	TMSB4X		
	3	3	FTH1P5	RPL39	ZNF721			
	4	5	FAU	GJA1	HBB	RPS29	SEPP1	
	5	7	DYNLL1	HMGN3	RPL30	RPL41	RPS11	RPS23
			UBB					
	6	6	FTL	HNRNPA1	RPS13	TPT1	RPL9	RPS18
	7	6	KIAA1598	YWHAQ	RPSA	RP11.403P17.4	TUBB2A	RTN1
	8	10	DPYSL2	FTH1	HAUS2	PFKFB3	RPL23A	RPL27
			RPS3A	ZNF160	RPS17	RPL34		
	9	8	CNP	GLTSCR2	HSPA1A	MAL	NACAP1	RNU86
			RPS24	RPS19				
	10	9	ANP32B	COX7A2	MT2A	RP5.882O7.1	RPS15	PAQR6
			TMA7	S100B	RPL32			

Supplementary Table 5. Results from 10 frontiers for MCO 1 thru MCO 4 pertaining to analysis instance 1-2D-7475

	Frontier	# of Genes	Gene Name					
MCO 1	1	2	HBA2	HBB				
	2	5	LOC100008589	LOC440589	OAZ1	RN7SL1	S100A9	
	3	3	FARSLB	LOC441876	RPS12			
	4	5	EEF1A1	LOC400721	RPS27	LOC646195	UBB	
	5	4	ARHGDIB	LOC645899	FTL	SLC25A39		
	6	5	ACTB	LOC389435	HBD	RPS2	TYROBP	
	7	7	LOC644039	LOC729603	LILRB3	LOC388720	CFD	LCP1
			PSAP					
	8	4	LOC643509	RPL19	RPL30	RPLP2		
	9	6	ORC6L	LOC285176	CSF3R	LOC399900	RPS3	UBA52
	10	5	CCL5	LOC652071	PDE4C	RPL27A	RPL39	
MCO 2	1	4	HBB	HBG1	HBG2	OAZ1		
	2	4	DEFA1	HBA2	LAIR1	RPL18A		
	3	5	ALPL	LOC646195	FCN1	RPS16	RPS27	
	4	6	GNB2L1	RNASET2	HBD	LITAF	GUK1	S100A8
	5	5	DEFA3	LYL1	RPL11	SEPX1	UBB	
	6	3	MYL6	TYROBP	UBC			
	7	6	C19orf31	CSDA	HLA.B	PSAP	RPS19	SRGN
	8	5	CFD	GLRX5	IL8RB	LOC728358	TMSB4X	
	9	7	EEF1A1	LOC401019	GYPC	LYN	RPS10	SLC25A39
			UBA52					
	10	7	ARHGDIB	LOC401206	F2R	LOC91561	MRCL3	RPS25
			SELL					
MCO 3	1	2	DEFA1	HBB				
	2	3	HBG1	HBG2	S100A9			
	3	2	HBA2	UBB				
	4	2	DEFA3	LAIR1				
	5	3	LOC388720	RPS4Y1	TYROBP			
	6	4	FTL	LOC728358	LITAF	RPS25		
	7	4	CST3	LOC441876	SEPX1	LOC642210		
	8	4	ACTB	BASP1	C19orf31	HLA.DRA		
	9	5	FARSLB	LOC652071	RPLP2	S100A8	TMSB4X	
	10	2	FCN1	RPS27				
MCO 4	1	1	HBB					
	2	2	HBA2	UBB				
	3	3	FCN1	LOC646195	S100A8			
	4	4	PSAP	LOC100008589	RNASET2	RPS4Y1		
	5	7	ALOX5AP	LOC400721	FARSLB	MX1	RN7SL1	RPS27
			RPS4X					
	6	6	CSF3R	LOC642210	FTL	MYL6	RPS10	TPT1
	7	5	HBG1	LOC645899	NCOA4	RPL18A	SNCA	
	8	5	ALPL	LOC729603	ORC6L	SLC25A39	PFN1	
	9	6	CYBA	LOC440589	GNB2L1	RNF213	RPL11	RPS25
	10	7	AQP9	HBG2	LCP1	LOC441876	LILRB3	RPL41
			ZNF486					

Supplementary Table 6. Results from 1 frontier for MCO 1 thru MCO 4 pertaining to analysis instance 3-3D-9039/8838/9587

	# of Genes	Gene Name						
MCO 1	26	ADM	ATP5E	CD74	CFL1	CORO1A	DYNLL1	FAM46C
		FLOT2	GPR97	IL4R	JUNB	MMP9	NADK	NINJ1
		PFDN5	PLEKHB1	RPS11	RPS24	SLC14A1	SLC4A1	TCIRG1
		TMEM140	TUBA1A	TUBA1B	TUBB2A	ZNF160		
MCO 2	39	AQP9	ARHGDIB	ARPP19	C5AR1	CEACAM4	CFL1	CORO1C
		DND1	FBXO7	FKBP8	FOS	FOSL2	GABARAP	GDI2
		GPX3	LSM3	MBP	MGAM	MMP9	NAMPT	NAPA
		NDRG1	NPRL3	NUDT21	PADI2	PI3	PTGDS	RAB5B
		RASSF2	RPL35A	RPS11	RPS23	RPS24	SIRPA	TRAK2
		TUBA1A	TUBA1B	TUBB2A	WLS			
MCO 3	33	ACTB	DDX3Y	MBP	PRKY	SLC48A1	TUBA1C	ADIPOR1
		EIF1AY	MS4A1	PTGDS	SRP14	VBP1	CCR1	FBXO7
		NPRL3	RPL30	TMEM176A	XAF1	CNP	FECH	PAQR6
		RPS24	TMEM176B	CSF1R	GZMK	PIP4K2A	RPS4X	TRAT1
		CYP4F3	ISCA1	PRKX	RPS4Y1	TUBA1A		
MCO 4	12	ATP5E	CFL1	DDX3Y	DYNLT3	HMGN3	PTPRO	RPS23
		RPS24	RPS29	RPS4Y1	UQCRB	VBP1		

Supplementary Table 7. Results from 1 frontier for MCO 1 thru MCO 4 pertaining to analysis instance 4-4D-9039/8838/9587/7475

	# of Genes	Gene Name						
MCO 1	87	ACTB	CEBPB	CST7	EEF2	GPR97	IFITM2	MMP9
		PADI4	PSMB3	RPL37A	RPS19	S100A8	SLC4A1	TMSB10
		TYROBP	ADM	CFD	DAZAP2	EMR3	HBB	IL17RA
		MYL6	PELI1	PTGDS	RPL41	RPS24	S100A9	SNCA
		TNS1	ZNF160	ALPL	CFL1	DUSP1	EPB42	HBD
		IL4R	NADK	PFDN5	RHOG	RPL8	RPS29	SELL
		TCIRG1	TUBA1A	ZYX	ARHGDIB	CORO1A	DYNLL1	FAM46C
		HCLS1	JUNB	NELL2	PI3	RPL11	RPL9	RPS4Y1
		SELPLG	TIMP1	TUBA1B	CD74	CSF3R	DYSF	FLOT2
		HLA.DPA1	LAPTM5	NINJ1	PLEKHB1	RPL18	RPS11	S100A11
		SH3BGRL3	TKT	TUBA1C	CD81	CST3	EEF1A1	GLRX5
		IFI27	LITAF	OAZ1	PSAP	RPL30	RPS15	S100A6
		SLC14A1	TMEM140	TUBB2A				
MCO 2	95	ACTB	C5AR1	CFD	DAZAP2	EIF1	FTL	GYPC
		ITM2B	LYZ	MYL6	OAZ1	PTGDS	RPL18	RPS23
		SUCLA2	TUBA1C	ALPL	CCNI	CFL1	DND1	FBXO7
		GABARAP	HBB	LAIR1	MARCKSL1	MYOM2	PADI2	RAB5B
		RPL27A	RPS24	TAGLN2	TUBB2A	AMFR	CD14	CNBP
		DPYSL2	FCN1	GDI2	HLA.B	LGALS1	MBP	NAMPT
		PI3	RASSF2	RPL35A	RPS6	TALDO1	TYROBP	AQP9
		CD74	CORO1C	DYNC1I2	FKBP8	GPX3	HLA.E	LILRB3
		MGAM	NAPA	PRF1	RGS2	RPL41	SIRPA	TRAK2
		UBB	ARHGDIB	CD81	CST3	EEF1A1	FOS	GPX4
		IFITM3	LITAF	MKLN1	NDRG1	PRKCD	RHOA	RPS11
		SORL1	TUBA1A	UBC	B4GALT5	CEACAM4	CSTA	EEF2
		FOSL2	GUK1	IL18R1	LSM3	MMP9	NUDT21	PSAP
		RPL11	RPS16	SRGN	TUBA1B			
MCO 3	108	ACTB	BAZ2B	CFL1	CYP4F3	FAM46C	GNS	HLA.E
		IL7R	LMBRD1	MRPL3	PI3	RHOA	RPL35A	RPS4X
		SLC7A5	SUB1	TRAT1	UBB	ADIPOR1	C14orf2	CHI3L1
		DDX3Y	FBXO7	GSPT1	HSDL2	ISCA1	LRRN3	MS4A1
		PJA2	RIOK3	RPS16	RPS4Y1	SOD1	TMED2	TRIM58
		UBC	AHCYL1	CAB39	CNP	DPM1	FECH	GZMB
		HSP90AA1	ITM2B	LTF	NAMPT	PRKX	RPL22	RPS18
		RPS6	SRGN	TMEM176A	TUBA1A	VBP1	ALPL	CCR1
		CSF1R	EIF1AX	FTL	GZMK	IFI16	KCNJ2	LYZ
		NDRG1	PRKY	RPL30	RPS24	S100A9	SRP14	TMEM176B
		TUBA1B	XAF1	AQP9	CCR7	CST3	EIF1AY	GCA
		HBB	IFITM1	LAIR1	MAL	NOSIP	PSAP	RPL31
		RPS25	SELL	SRP19	TMSB10	TUBA1C	YWHAZ	BASP1
		CD74	CYBRD1	EMR2	GMPR	HBZ	IFITM2	LITAF
		MBP	PAQR6	PTGDS	RPL35	RPS29	SLC4A1	STK4
		TMSB4X	TYROBP	ZFAND5				
MCO 4	31	ANP32B	ATP6V1E1	B2M	CFL1	DDX3Y	DYNLT3	EEF1A1
		HBB	HMGN3	MAL	OAZ1	PTGDS	PTPRO	RPL11
		RPL22	RPL30	RPL35	RPL41	RPL6	RPS16	RPS18
		RPS19	RPS23	RPS24	RPS25	RPS29	RPS4X	RPS4Y1
		RPS6	S100A8	TBCA				

References

1. Hall MA. Correlation-based feature selection for machine learning. PhD Thesis, Waikato University. 1999.

2. Hall MA and Smith LA. Practical feature subset selection for machine learning. In Proceedings of the 21st Australasian Computer Science Conference. 1998:98,181–191.

3. Smyth GK. Linear models and empirical bayes methods for assessing differential expression in microarray experiments. Statistical applications in genetics and molecular biology. 2004:3(1).

4. Singhal S and Jena M. A Study on WEKA Tool for Data Preprocessing , Classification and Clustering. International Journal of Innovative technology and exploring engineering (IJItee). 2013:2(6),250-253

5. Romanski P and L. Kotthoff L. Package FSelector: Selecting Attributes. URL http://cran/r-project. org/web/packages/FSelector/index html. 2013.

6. Ritchie ME, Phipson B, Wu D, Hu Y, Law CW, Shi W, Smyth GK. limma powers differential expression analyses for RNA-sequencing and microarray studies. Nucleic Acids Research 2015:43(7),e47.

7. Cortes C and Vapnik V. Support-Vector Networks. Machine learning. 1995:20(3),273-297.

8. Cover T and Hart P. Nearest neighbor pattern classification. IEEE transactions on information theory. 1967:13(1),21-27.

9. Breiman L. Bagging predictors. Machine Learning. 1996:24(2),123–140.

10. Fisher RA. The Use of Multiple Measurements in Taxonomic Problems. Annals of Eugenics. 1936:7(2),179–188.

11. Breiman L. Random Forests. Machine Learning. 2001:45(1),5–32.

12. Kuhn M. Caret package. Journal of Statistical Software. 2008:28(5).

13. Girden ER. (1992). ANOVA Repeated measures. Sage 1992:84.

14. Benjamini Y, and Braun H. John W. Tukey's contributions to multiple comparisons. Annals of Statistics. 2002:1576-1594.

15. Levene H. Robust tests for equality of variances. Contributions to Probability and Statistics: Essays in Honor of Harold Hotelling. S1960:278–292.
